# Supplementary material for: Characteristics of ferroelectric-ferroelastic domains in Néel-type skyrmion host GaV4S8
Source: Sci Rep. 2017 Mar 15;7:44663. doi: 10.1038/srep44663 (PMC5353724; doi:10.1038/srep44663)
Supplement: Supplementary Information [file srep44663-s1.pdf]

Characteristics of ferroelectric-ferroelastic domains in Néel-type skyrmion host GaV_4S_8

Ádám Butykai[1,*], Sándor Bordács[1], István Kézsmárki[1], Vladimir Tsurkan[2,3], Alois Loidl[2], Jonathan Döring[4], Erik Neuber[4], Peter Milde[4], Susanne C. Kehr[4], and Lukas M. Eng[4,5]

[1]Department of Physics, Budapest University of Technology and Economics and MTA-BME Lendület Magneto-optical Spectroscopy Research Group, 1111 Budapest, Hungary
[2]Experimental Physics V, Center for Electronic Correlations and Magnetism, University of Augsburg, 86135 Augsburg, Germany
[3]Institute of Applied Physics, Academy of Sciences of Moldova, MD 2028, Chisinau, Republica Moldova
[4]Institut für Angewandte Physik, Technische Universität Dresden, D-01062 Dresden, Germany
[5]Center for Advancing Electronics Dresden cfaed, Technische Universität Dresden, 01062 Dresden, Germany

1 PFM contrast

In this section, we describe the piezo-response of GaV_4S_8 in its cubic and rhombohedral phase, using symmetry considerations, in order to establish the PFM contrast between the structural variants of the crystal.

Neumann's principle has been applied to obtain the structure of the inverse piezoelectric tensor for the two inversion variants of the crystal in the high-temperature cubic phase, as presented in Eqs. 1. The base vectors of the coordinate system, $\{\mathbf{e}_x, \mathbf{e}_y, \mathbf{e}_z\}$, point along the cubic [100], [010] and [001] directions, respectively. The tensors expressed in the Voigt-notation read as:

$$d_{A,c}^{[001]} = \begin{pmatrix} 0 & 0 & 0 \\ 0 & 0 & 0 \\ 0 & 0 & 0 \\ 0 & d & 0 \\ d & 0 & 0 \\ 0 & 0 & d \end{pmatrix}, \qquad d_{B,c}^{[001]} = \begin{pmatrix} 0 & 0 & 0 \\ 0 & 0 & 0 \\ 0 & 0 & 0 \\ 0 & -d & 0 \\ -d & 0 & 0 \\ 0 & 0 & -d \end{pmatrix}, \tag{1}$$

where A and B indices denote the two inversion variants and the label 'c' refers to the cubic phase of the compound.

The non-centrosymmetric structure of GaV_4S_8 is responsible for the finite piezo-response, represented by the non-vanishing tensor elements with magnitudes of d and $-d$ in the A and B variants, respectively.

The out-of-plane PFM measurement probes the d_{zzz} element of the inverse piezoelectric tensor, where the z-direction is normal to the scanned surface. When $z = [001]$, that is, the (001) surface is scanned, the piezo-response vanishes, i.e. $d_{zzz,c}^{[001]} = 0$, which is in accord with the structureless PFM images recorded above T_s [see Fig. S1].

In order to obtain the magnitude of the piezo-response probed by PFM measurements on the (111) surface of the crystal, i.e. for $z = [111]$, the d_{zzz} tensor element is expressed in the coordinate system of the PFM tip, defined by: $\mathbf{e'_x} = 1/\sqrt{2}(10\bar{1})$, $\mathbf{e'_y} = 1/\sqrt{6}(1\bar{2}1)$ and $\mathbf{e'_z} = 1/\sqrt{3}(111)$. Note that the new $\mathbf{e'_z}$ base vector is normal to the scanned (111) plane, whereas $\mathbf{e'_x}$ and $\mathbf{e'_y}$ orthogonal base vectors may be chosen arbitrarily within the (111)-plane without affecting the d_{zzz}-component. The transformation yields $d_{zzz,c}^{[111]} = d_{xyz}^{[001]} = \pm d$, for variants A and B, respectively. Hence, inversion domain boundaries may be visualized by PFM measurements on the (111) surface in the cubic phase of the compound. Nevertheless, no PFM images captured above the structural phase transition temperature reveal any contrast [see Fig. S1], suggesting that no inversion domains were present in the scanned surface areas.

In the rhombohedral phase, the symmetry-allowed components of the converse piezoelectric tensor have been determined for the four possible directions of the structural distortion, as presented in Eqs. 2. Here, domains originating from a single inversion variant (A) were considered. The corresponding piezoelectric tensors in the antiphase domains can be obtained by a

sign reversal. All the matrices can be described by four independent elements, d_1, d_2, d_3 and d_4.

$$d^{[001]}_{[111],r} = \begin{pmatrix} d_1 & d_2 & d_2 \\ d_2 & d_1 & d_2 \\ d_2 & d_2 & d_1 \\ d_3 & d_0 & d_3 \\ d_0 & d_3 & d_3 \\ d_3 & d_3 & d_0 \end{pmatrix}, \quad d^{[001]}_{[\bar{1}1\bar{1}],r} = \begin{pmatrix} -d_1 & d_2 & -d_2 \\ -d_2 & d_1 & -d_2 \\ -d_2 & d_2 & -d_1 \\ -d_3 & d_0 & -d_3 \\ d_0 & -d_3 & d_3 \\ d_3 & -d_3 & d_0 \end{pmatrix}, \quad d^{[001]}_{[\bar{1}\bar{1}1],r} = \begin{pmatrix} -d_1 & -d_2 & d_2 \\ -d_2 & -d_1 & d_2 \\ -d_2 & -d_2 & d_1 \\ d_3 & d_0 & -d_3 \\ d_0 & d_3 & -d_3 \\ -d_3 & -d_3 & d_0 \end{pmatrix}, \quad d^{[001]}_{[1\bar{1}\bar{1}],r} = \begin{pmatrix} d_1 & -d_2 & -d_2 \\ d_2 & -d_1 & -d_2 \\ d_2 & -d_2 & -d_1 \\ -d_3 & d_0 & d_3 \\ d_0 & -d_3 & -d_3 \\ -d_3 & d_3 & d_0 \end{pmatrix}. \tag{2}$$

In case of PFM measurements on the (001) plane, the probed tensor component reads: $d^{[001]}_{zzz,r} = d_1$ for domains [111] and $[\bar{1}\bar{1}1]$, whereas the other two domains, $[\bar{1}1\bar{1}]$ and $[1\bar{1}\bar{1}]$, exhibit a piezo-response with the same magnitude and an opposite sign, $d^{[001]}_{zzz,r} = -d_1$. Apparently, the structural distortion gives rise to a finite piezo-response in each structural variant, however, PFM contrast appears only between domains with an opposite z-component in their dielectric polarization vector. Indeed, the domains within both groups can be interchanged by a twofold rotation around the z-axis, leaving the d_{zzz} tensor element invariant, while point group transformations that interchange the two pairs of domains reverse the sign of d_{zzz}.

The $d^{[111]}_{zzz,r}$ tensor element probed in (111) plane PFM measurements are expressed for the four domains via the base transformation from $\{\mathbf{e}_x, \mathbf{e}_y, \mathbf{e}_z\}$ to $\{\mathbf{e}'_x, \mathbf{e}'_y, \mathbf{e}'_z\}$ as done previously in the cubic case:

$$\mathrm{d}^{[111]}_{zzz,r} = \begin{cases} 2\sqrt{3}/3 \cdot d_0 + \sqrt{3}/3 \cdot d_1 + 2\sqrt{3}/3 \cdot d_2 + 4\sqrt{3}/3 \cdot d_3, \\ \text{ for domain } [111] \\ 2\sqrt{3}/3 \cdot d_0 - \sqrt{3}/9 \cdot d_1 - 2\sqrt{3}/9 \cdot d_2 - 4\sqrt{3}/9 \cdot d_3, \\ \text{ for domains } [1\bar{1}\bar{1}], [\bar{1}1\bar{1}], [\bar{1}\bar{1}1]. \end{cases} \tag{3}$$

Remarkably, the magnitude of the probed piezo-response of the unique [111]-domain and the three other domains are not symmetric to zero, as opposed to PFM measurements in the (001) plane. Instead, the contrast is distributed around a constant baseline, $2\sqrt(3)/3d_0$, with a ratio of 3:1 for the [111]-domain and the other three domains, respectively. Note that in the antiphase variants, the sign of the probed tensor element is reversed, including that of the baseline term. Therefore, the presence of inversion variants must be reflected in the PFM contrast measured on a (111) surface in the rhombohedral phase, which does not vanish above the structural phase transition. Nevertheless, such antiphase domains have not been evidenced throughout our measurements.

2 Collection of all PFM measurements

Figure 1 presents all the measurements where PFM contrast has been observed in the rhombohedral phase of GaV_4S_8. All images display the raw PFM amplitude data without baseline correction. PFM images in the same row represent measurements near the same location. Red squares indicate the scanned area in the subsequent image. PFM scans obtained above the structural phase transition temperature, i.e. in the cubic phase of the crystal, are presented in the right column of the figure. The PFM images taken in the cubic phase are aligned in the same row with the ones in the rhombohedral phase, whenever they were captured over the same surface area.

The PFM images in the rhombohedral phase have been analyzed via 2D-Fourier transformation to extract the typical periodicity of the domains. The width of the domain walls were determined as $w_i = 2\pi/|k_i|$, where $|k_i|$ represents the locations of the most dominant peaks in the two-dimensional Fourier-space. In case of measurements on the (111) surface, the projection of the (100)-type domain walls are detected. Therefore, the measured domain widths have been scaled down by a factor of $\sqrt{3}$ to obtain the actual domain spacings in the (100) plane.

3 Calculating the surface inclination angles

In this section we calculate the inclination angles between the distorted (100) and (111) surfaces of two adjoining compatible rhombohedral domains in GaV_4S_8. Pocha *et al.*[1] report a rhombohedral angle in GaV_4S_8 of 59.66° at 20 K, based on X-ray scattering data. Figure 2 (a) displays a rhombohedrally distorted cube along the [111]-direction in the Cartesian coordinate system, representing a [111]-type rhombohedral domain. In Fig. 2 (b) the same [111]-domain is presented together with a $[\bar{1}\bar{1}1]$-type domain. Mechanical and electric compatibility requires that these two domains match on the (001) plane[2], i.e. the (x,y)-plane of our coordinate system.

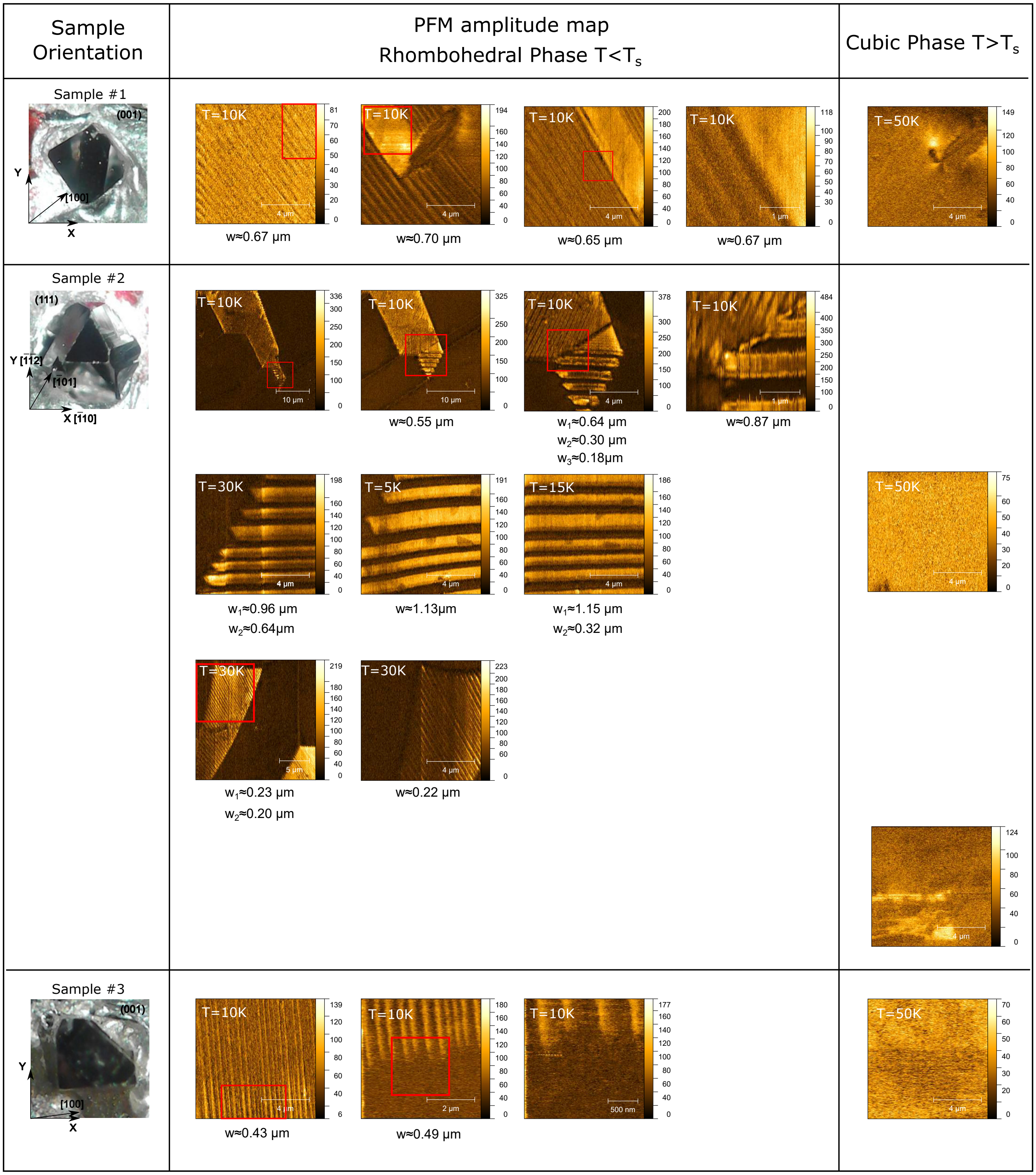

Figure 1. Collection of all PFM amplitude images, where significant contrast was found in the rhombohedral phase of GaV_4S_8. Images in the same row present subsequent PFM measurements over the same surface area of the crystal. Red rectangles represent the scanned area of the subsequent PFM image. PFM images showing no contrast in the cubic phase of the crystal are also presented in the right column. The approximate domain widths, determined by 2D-Fourier transformation of the PFM amplitude images, are listed below the images. In case of the measurements in the (111) plane of Sample #2, the measured domain widths have been scaled down by a factor of $\sqrt{3}$.

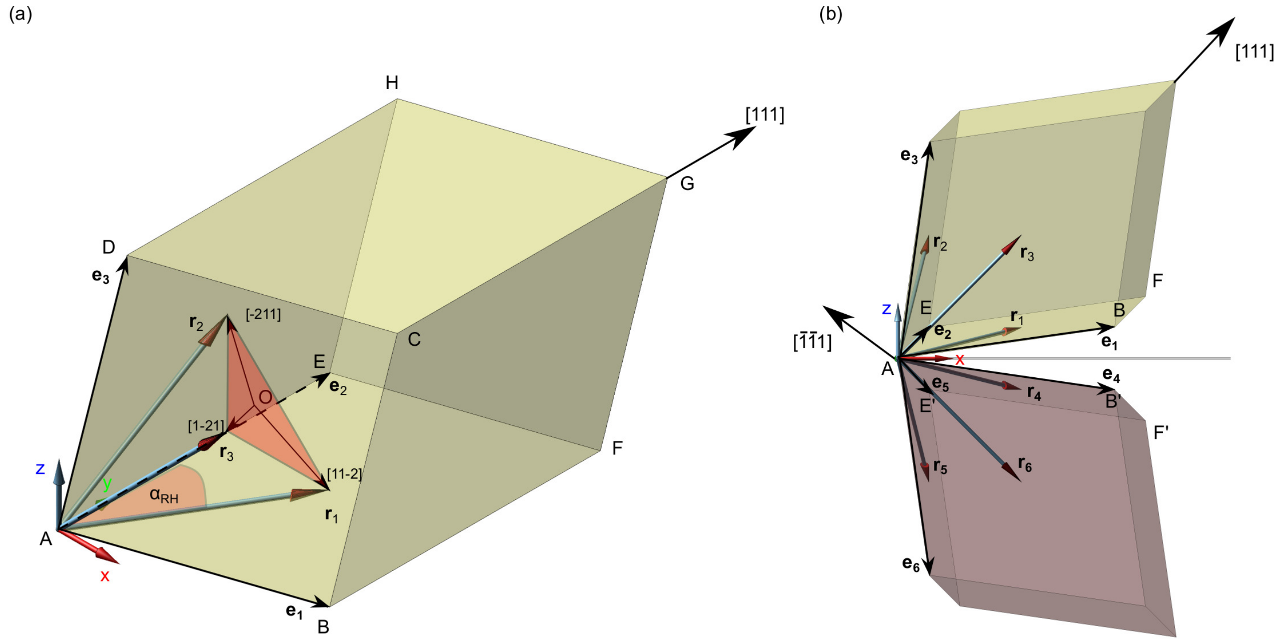

Figure 2. Panel (a) shows a rhombohedrally distorted cube along the [111]-axis, representing a [111]-domain in GaV_4S_8. Panel (b) displays two cubes distorted along [111] and $[\bar{1}\bar{1}1]$ looking from the (x,z)-plane. Mechanical and electric compatibility holds for an interface with (001)-normal, i.e. the (x,y)-plane. The orthogonal transformations described in the text match the A,B,F,E corners of the top cube with the A,B',F',E' corners of the bottom cube, by rotating them into the (x,y)-plane.

As a result of the distortion, the edges of the two rhombohedra, $\{\mathbf{e}_1, \mathbf{e}_2, \mathbf{e}_3\}$ deviate from the cubic coordinate system, $\{x, y, z\}$. Our goal is to match the corners of the two rhombohedra, $\{A, B, F, E\}$ with $\{A, B', F', E'\}$ by rotating them into the (x, y)-plane in order to ensure the continuouity of the lattice on the two sides. To achieve that, we shall express the $\{\mathbf{e}_1, ..., \mathbf{e}_3\}$ vectors in terms of the rhombohedral angle and the axes of the distortions.

The axis of the rhombohedral stretching and the plane perpendicular to this axis [red triangle in Fig. 2 (a)] constitute eigenspaces of the distortion. Using the invariant [111] and $[11\bar{2}]$-type directions [indicated by black arrows starting from point O in Fig. 2 (a)], the $\{\mathbf{r}_1, ..., \mathbf{r}_6\}$ base vectors in the rhombohedral system can be expressed as:

$$
\begin{aligned}
\mathbf{r}_1 &= c_{hex}\frac{1}{3\sqrt{3}} * [111] + \frac{2}{3}\frac{\sqrt{3}}{2}a_{hex}\frac{1}{\sqrt{6}} * [11\bar{2}], & \mathbf{r}_4 &= c_{hex}\frac{1}{3\sqrt{3}} * [11\bar{1}] + \frac{2}{3}\frac{\sqrt{3}}{2}a_{hex}\frac{1}{\sqrt{6}} * [112], \\
\mathbf{r}_2 &= c_{hex}\frac{1}{3\sqrt{3}} * [111] + \frac{2}{3}\frac{\sqrt{3}}{2}a_{hex}\frac{1}{\sqrt{6}} * [\bar{2}11], & \mathbf{r}_5 &= c_{hex}\frac{1}{3\sqrt{3}} * [11\bar{1}] + \frac{2}{3}\frac{\sqrt{3}}{2}a_{hex}\frac{1}{\sqrt{6}} * [\bar{2}1\bar{1}], \\
\mathbf{r}_3 &= c_{hex}\frac{1}{3\sqrt{3}} * [111] + \frac{2}{3}\frac{\sqrt{3}}{2}a_{hex}\frac{1}{\sqrt{6}} * [1\bar{2}1], & \mathbf{r}_6 &= c_{hex}\frac{1}{3\sqrt{3}} * [11\bar{1}] + \frac{2}{3}\frac{\sqrt{3}}{2}a_{hex}\frac{1}{\sqrt{6}} * [1\bar{2}\bar{1}],
\end{aligned} \tag{4}
$$

where a_{hex} and c_{hex} are the lengths of the base vectors in the equivalent hexagonal crystal system: $a_{hex} = 2\sin\alpha_{RH}/2$ and $c_{hex} = 3\sqrt{4/3\cos^2\alpha_{RH}/2 - 1/3}$, α_{RH} is the rhombohedral angle known from x-ray diffraction data. The unit vectors pointing along the three edges of the rhombohedrons read as:

$$
\begin{aligned}
\mathbf{e}_1 &= (\mathbf{r}_1 - \mathbf{r}_2 + \mathbf{r}_3)/\left\|\mathbf{r}_1 - \mathbf{r}_2 + \mathbf{r}_3\right\| & \mathbf{e}_4 &= (\mathbf{r}_4 - \mathbf{r}_5 + \mathbf{r}_6)/\left\|\mathbf{r}_4 - \mathbf{r}_5 + \mathbf{r}_6\right\| \\
\mathbf{e}_2 &= (\mathbf{r}_1 + \mathbf{r}_2 - \mathbf{r}_3)/\left\|\mathbf{r}_1 + \mathbf{r}_2 - \mathbf{r}_3\right\| & \mathbf{e}_5 &= (\mathbf{r}_4 + \mathbf{r}_5 - \mathbf{r}_6)/\left\|\mathbf{r}_4 + \mathbf{r}_5 - \mathbf{r}_6\right\| \\
\mathbf{e}_3 &= (-\mathbf{r}_1 + \mathbf{r}_2 + \mathbf{r}_3)/\left\|-\mathbf{r}_1 + \mathbf{r}_2 + \mathbf{r}_3\right\| & \mathbf{e}_6 &= (-\mathbf{r}_4 + \mathbf{r}_5 + \mathbf{r}_6)/\left\|-\mathbf{r}_4 + \mathbf{r}_5 + \mathbf{r}_6\right\|
\end{aligned} \tag{5}
$$

Now, we define two rotation matrices to align $\mathbf{e}_1$ and $\mathbf{e}_4$ with x, $\mathbf{e}_1 \times \mathbf{e}_2$ and $\mathbf{e}_4 \times \mathbf{e}_5$ with z, and the cross-product of the first

two vectors in both domains with y:

$$R_1 = \begin{bmatrix} \mathbf{e}_1, & \frac{(\mathbf{e}_1\times\mathbf{e}_2)\times\mathbf{e}_1}{\|(\mathbf{e}_1\times\mathbf{e}_2)\times\mathbf{e}_1\|}, & \frac{\mathbf{e}_1\times\mathbf{e}_2}{\|\mathbf{e}_1\times\mathbf{e}_2\|} \end{bmatrix}^T$$
$$R_2 = \begin{bmatrix} \mathbf{e}_4, & \frac{(\mathbf{e}_4\times\mathbf{e}_5)\times\mathbf{e}_4}{\|(\mathbf{e}_4\times\mathbf{e}_5)\times\mathbf{e}_4\|}, & \frac{\mathbf{e}_5\times\mathbf{e}_5}{\|\mathbf{e}_4\times\mathbf{e}_5\|} \end{bmatrix}^T \tag{6}$$

The (010) and (111) surface normals are expressed in the coordinate system of the two distorted rhombohedrons:

$$\begin{aligned} n^{[111]}_{(010)} &= \frac{\mathbf{e}_1 \times \mathbf{e}_3}{\|\mathbf{e}_1 \times \mathbf{e}_3\|}, \\ n^{[111]}_{(111)} &= \frac{1}{\sqrt{3}}[111], \\ n^{[\bar{1}\bar{1}1]}_{(010)} &= \frac{\mathbf{e}_4 \times \mathbf{e}_6}{\|\mathbf{e}_4 \times \mathbf{e}_6\|}, \\ n^{[\bar{1}\bar{1}1]}_{(111)} &= \frac{(\mathbf{e}_5 + \mathbf{e}_6) \times (\mathbf{e}_5 - \mathbf{e}_4)}{\|(\mathbf{e}_5 + \mathbf{e}_6) \times (\mathbf{e}_5 - \mathbf{e}_4)\|}, \end{aligned} \tag{7}$$

where the lower indices in round parentheses refer to the surfaces in the neighboring domains and the upper indices in squared parentheses denote the axis of distortion in the given domain.

Finally, we transform the normal vectors of the neighboring surfaces to the Cartesian coordinate system and calculate the inclination angles:

$$\begin{aligned} \gamma_{[010]} &= arccos(R_1 n^{[111]}_{(010)} \cdot R_2 n^{[\bar{1}\bar{1}1]}_{(010)}), \\ \gamma_{[111]} &= arccos(R_1 n^{[111]}_{(111)} \cdot R_2 n^{[\bar{1}\bar{1}1]}_{(111)}). \end{aligned} \tag{8}$$

Substituting $\alpha_{RH} = 59.66^\circ$ into the above calculation yields $\gamma_{[010]} = 0.5844^\circ$ and $\gamma_{[111]} = 0.5515^\circ$.

References

1. Pocha, R., Johrendt, D. & Pöttgen, R. Electronic and structural instabilities in gav4s8 and gamo4s8. *Chemistry of materials* **12**, 2882–2887 (2000).

2. Shu, Y. & Bhattacharya, K. Domain patterns and macroscopic behaviour of ferroelectric materials. *Philosophical Magazine B* **81**, 2021–2054 (2001).
